# Association and Correlation Between Amniotic Fluid Index and Glucose Concentration

**DOI:** 10.7759/cureus.25973

**Published:** 2022-06-15

**Authors:** Sachin Khanduri, Harleen Chawla, Asif Khan, Surbhi LNU, Vaibhav Pathak, Ashkrit Gupta, Juned Shaikh, Sana Fatima, Zaara Khan, Vasundhra LNU

**Affiliations:** 1 Radiology, Era's Lucknow Medical College and Hospital, Lucknow, IND; 2 Radiodiagnosis, Era’s Lucknow Medical College and Hospital, Lucknow, IND; 3 Radiodiagnosis, Era's Lucknow Medical College and Hospital, Lucknow, IND

**Keywords:** avoiding oral intake of sugar, gestational diabetes, serum glucose concentration, polyhydramnios, amniotic fluid index

## Abstract

Purpose: To study the association and correlation between the amniotic fluid index, random glucose concentration, and serum glucose concentration after avoiding oral intake of sugar in a pregnant female with polyhydramnios.

Methods: The research was performed on pregnant women with polyhydramnios (n=104 ) after 28 weeks. USG was performed using a SAMSUNG HS 70A (Samsung Electronics Pvt. Ltd., Seoul, South Korea) and a GE Voluson P8 (GE Healthcare, Little Chalfont, UK). We measured amniotic fluid index and took a blood sample for hemoglobin (Hb)A1C, fasting blood glucose, post-prandial and random blood glucose, and also performed a glucose tolerance test in pregnant women.

Results: This is a prospective study, all 104 patients that were recruited in this study were pregnant females with polyhydramnios mainly from the urban and rural zone with different age groups (between 21 and 37 years). In our study, we observed that after avoiding oral intake of sugar in pregnant females with polyhydramnios, it was concluded that the amnionic fluid index lies towards the lower side. Polyhydramnios is more common in the urban zone and among older pregnant females. Out of 104 pregnant females with polyhydramnios, 82 were diagnosed with gestational diabetes after 28 weeks.

Conclusion: In this study, we have concluded that the earliest and most sensitive predictor for gestational diabetes is a rise in the amniotic fluid index which could have been prevented by avoiding oral intake of sugar. Early prediction of gestational diabetes can be made by amniotic fluid index even before glucose concentration. We observed that by reducing oral intake of sugar, the amniotic fluid index drops down in pregnant females

## Introduction

There is a well-defined affiliation between diabetes mellitus and pregnancy complications through hydramnios [1]. Approximately 15% of pregnancies with hydramnios arise amongst ladies with diabetes [2,3]. Polyhydramnios is described as a pathological growth of amniotic fluid extent in being pregnant and is related to accelerated perinatal morbidity and mortality. Common reasons for polyhydramnios encompass gestational diabetes, fetal anomalies with disturbed fetal swallowing of amniotic fluid, fetal infections, and other, rarer reasons. The analysis is acquired through ultrasound. The diagnosis of polyhydramnios relies upon its reason and severity. Typical signs of polyhydramnios encompass maternal dyspnea, early labor, untimely rupture of membranes (PPROM), bizarre fetal presentation, cord prolapse, and postpartum hemorrhage [4].

Under physiological conditions, there may be a dynamic equilibrium between the production and resorption of amniotic fluid. Fluids are stimulated through fetal urination and fetal lung liquid manufacturing. Amniotic fluid is reabsorbed through fetal swallowing and intramembranous and intravascular absorption. The relative attribution of every one of those mechanisms varies all through being pregnant. A disturbed equilibrium may be the result of compromised swallowing features or accelerated urination and might cause polyhydramnios [5-8].

Ultrasound assessment of amniotic fluid volume

The assessment of amniotic fluid at some point of an ordinary ultrasound test may be executed in three ways: the subjective way, the dimension of the single biggest vertical pocket, or the assessment of the amniotic fluid index. The subjective technique includes visually estimating the amniotic fluid pockets at the same point of an ultrasound examination. The accuracy of this evaluation will depend strongly on the experience of the sonographer [9]. The biggest vertical pocket assessment includes measuring the biggest pocket freed from the fetal systems or a cord with the ultrasound probe located parallel to the sagittal plane [10,11]. 

## Materials and methods

This is a prospective study, the total 104 patients that were recruited in this study were pregnant females with polyhydramnios from the urban and rural zone. The study was carried out at the department of Radio-diagnosis in collaboration with the Department of Obstetrics and Gynaecology, Era’s Lucknow Medical College from September 2020 to September 2021 after getting clearance from the Institutional Ethical Committee (approval no. ELMC & H /RCELL, EC/2021/132). Informed consent from the patients was taken. All the examinations were carried out under ethical standards which were approved by the Declaration of Helsinki in 1964, and its revisions occurred due to the course of time.

USG was performed using a SAMSUNG HS 70A (Samsung Electronics Pvt. Ltd., Seoul, South Korea ) and GE Voluson P8 (GE Healthcare, Little Chalfont, United Kingdom) ultrasonography machine with a 5MHz curvilinear electronic array. Two radiologists (with more than five years of experience) reviewed the ultrasonography.

Inclusion criteria: Pregnant females with singletons and polyhydramnios with more than 21 weeks of gestational age. Exclusion criteria: Twin pregnancy and multiple pregnancies, known case of diabetes, pregnant females with medication history of drugs related to carbohydrate metabolism. For all patients who fulfilled the above-mentioned criteria, random blood sugar was taken and afterward, they were asked to avoid oral intake of sugar until delivery, then a series of ultrasonography was performed at 28 weeks followed by 30 weeks, 32 weeks then at 36 weeks, and finally at term.

Statistical analysis

Considering P=15%, Z= 95% CI and E=7%, the sample size calculation with prevalence was done as follows: n = Zα/22 *P*(1-P) /E^2^ = 4*15*(100-15)/7^2^ to give n=104. Where P is the prevalence, Z is the confidence limit, E is the margin of error, n is the sample size and CI is the confidence interval.

The research was performed in pregnant women with polyhydramnios (n=104), We measured amnionic fluid index, and HbA1C in the serum of pregnant women. Glucose tolerance test, blood glucose fasting, post-prandial and random blood glucose in pregnant women.

## Results

**Figure 1:**
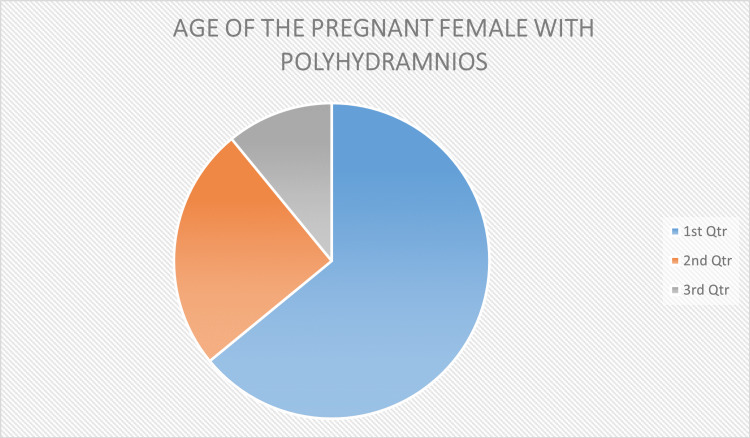
Age of the pregnant female with polyhydramnios 1st Qtr: Age of the pregnant female 34-37 years; 2nd Qtr: Age of the pregnant female 28 -34 years; 3rd Qtr: Age of the pregnant female 21-27 years

The total 104 patients that were recruited in this study were pregnant females with polyhydramnios from the urban and rural zone with different age groups (between 21 and 37 years). All women fulfilled the inclusion criteria. In our study, we observed that after avoiding oral intake of sugar in pregnant females with polyhydramnios, the amnionic fluid index dropped towards the lower side. Out of 104 pregnant females with polyhydramnios, 82 were diagnosed with gestational diabetes. The study depicts the relationship between the amniotic fluid index and blood sugar levels in pregnancies. The pie chart (Figure 1) is showing that out of total females about 64 percent of pregnant females had an age greater than 34 years, 25 percent of females were in the range of 28-34 years and 11 percent were falling in the range of 21-27 years.

**Figure 2:**
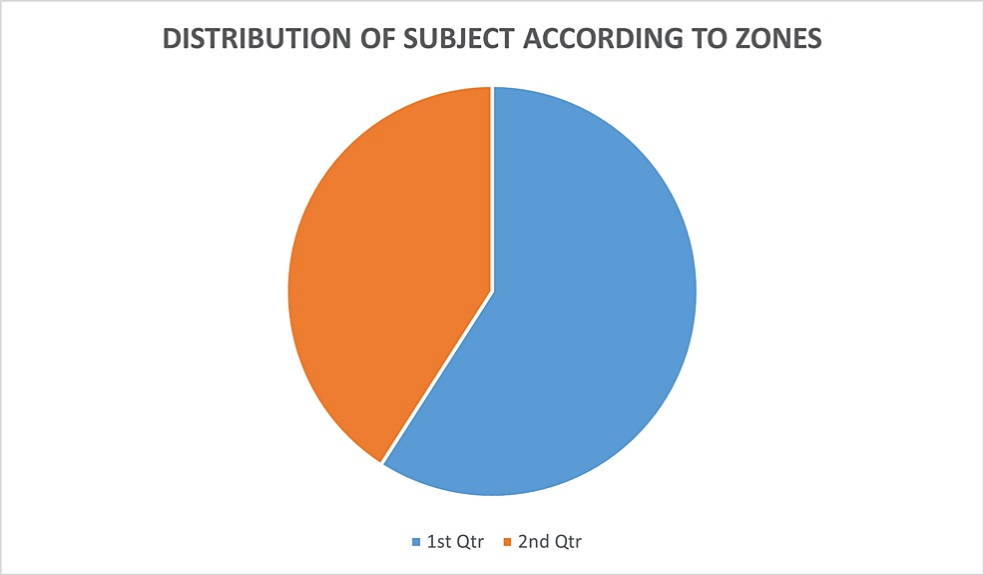
Distribution of subjects according to zones: Urban sector and Rural sector 1st Qtr: Urban area - 55%; 2nd Qtr: Rural area - 45%

The pie chart in Figure 2 is showing that out of total females about 55 percent belonged to the urban sector and the rest 45 percent were from the rural sector. 

**Figure 3:**
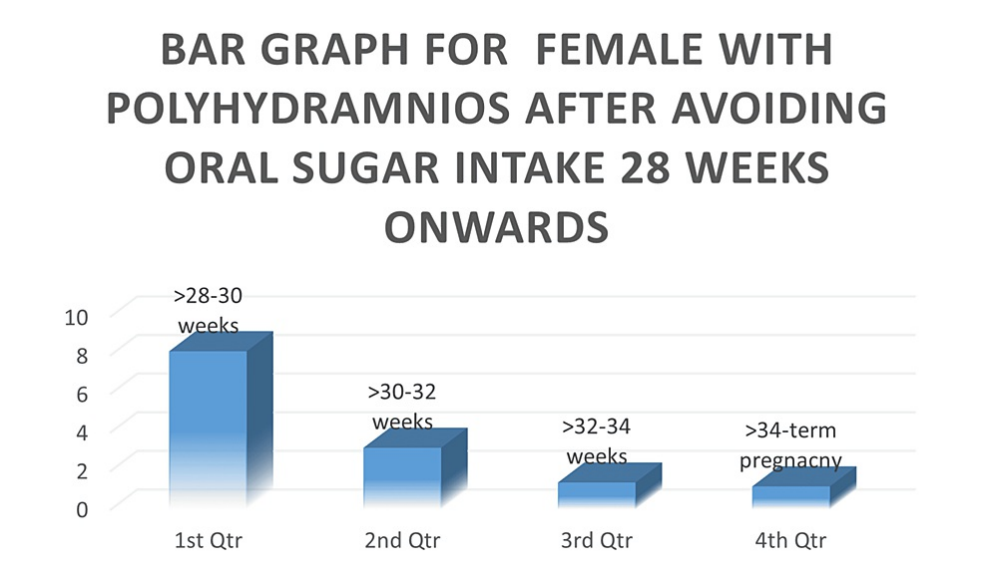
Bar graph for females with polyhydramnios after avoiding oral sugar intake 28 weeks onwards Avoiding oral intake of sugar, the amniotic fluid index drops down in pregnant females with polyhydramnios.

We observed in the following bar graph (Figure 3) that females with polyhydramnios after avoiding oral sugar intake beyond 28 weeks decreased as the gestational age was increased to the extent that more than 75 percent of females showed a decrease in their amniotic fluid index from there original values. Also, the change was more rapid in the gestational age range of 28-34 weeks, whereas beyond 34 weeks the values stabilized.

We measured amnionic fluid index (Table 1) and HbA1C in the serum of pregnant women. Glucose tolerance test, blood glucose fasting, post-prandial and random blood glucose in pregnant women (Table 2).

**Table 1:** USG findings at 28 weeks.

No. of Pregnant females with Polyhydramnios	More than 50 Percentile of Amniotic Fluid Index
25	22.0 -23.9
24	24.0 -25.9
20	26.0 -27.9
20	28.0 -29.9
15	30.0 -31.0

**Table 2:** Laboratory tests at 28 weeks of gestational age Hemoglobin (Hb)A1C in the serum of pregnant women, glucose tolerance test, blood glucose fasting, post-prandial and random blood glucose in pregnant women at 28 weeks of gestational age.

LAB TEST	No. of subjects	Result
HbA1C in serum of pregnant women	10	5.6-6.4 %(prediabetic )
Glucose tolerance test in pregnant women	65	>200 mg/dl
Blood glucose fasting in pregnant women	65	>125 mg/dl
Post-prandial blood glucose in pregnant women	75	>199 mg/dl
Random blood glucose in pregnant women	85	>200 mg/dl

Image analysis

**Figure 4:**
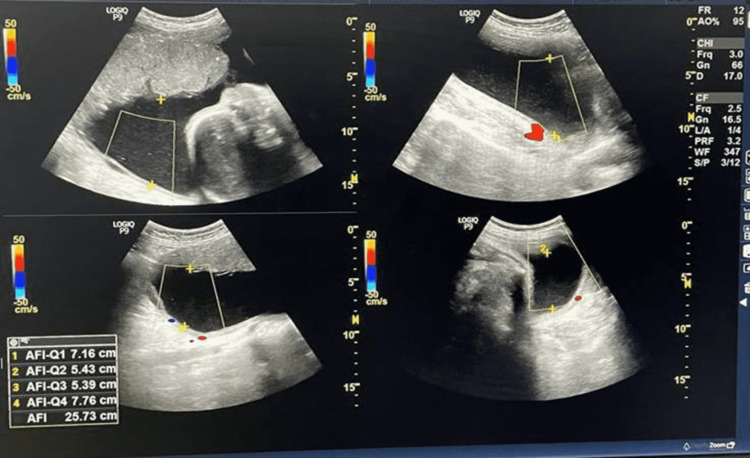
USG showing AFI at 28 weeks of gestation for the first patient. Amniotic fluid index (AFI) = 25.7cm The patient was advised to restrict her intake of oral sugar and blood samples were taken for analysis.

**Figure 5:**
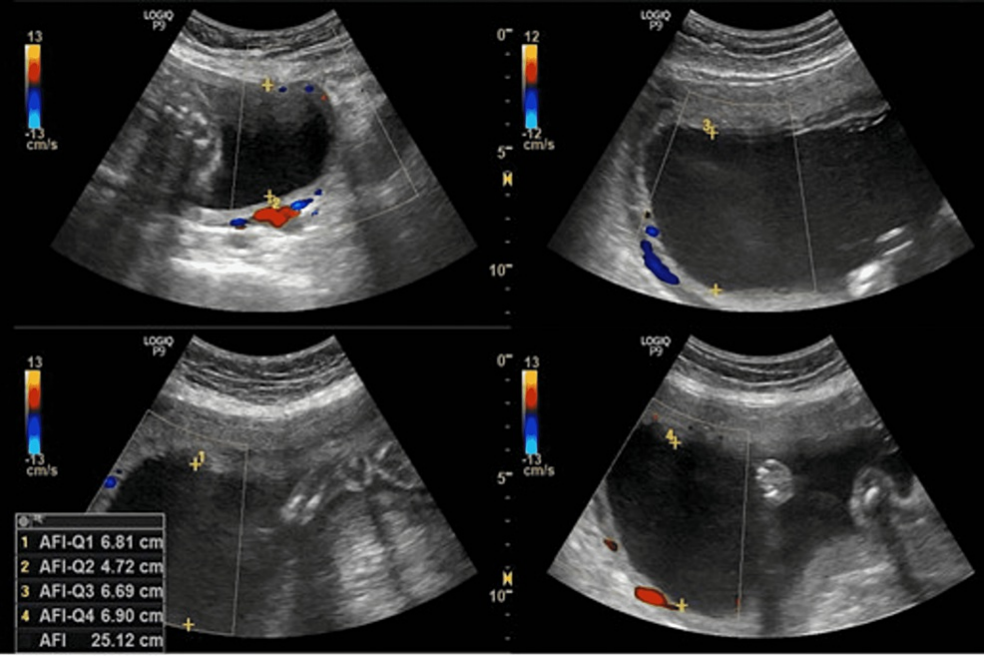
USG image showing AFI at 32 weeks of gestation for the first patient. Amniotic fluid index (AFI) = 25.1cm

**Figure 6:**
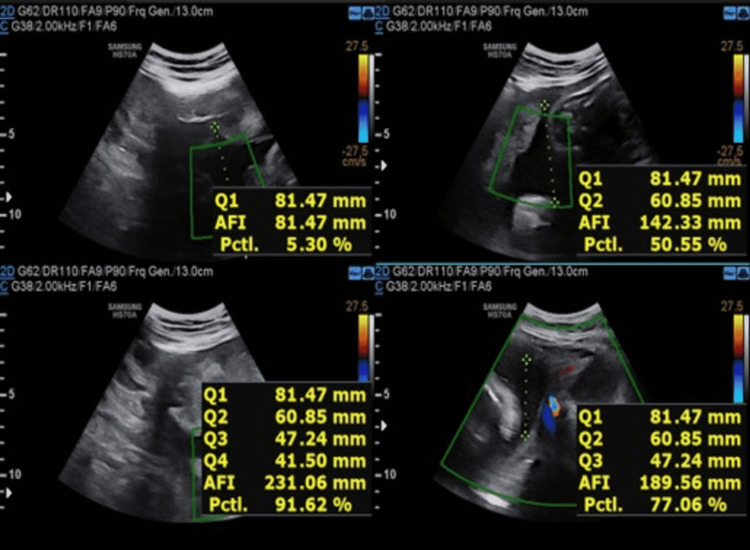
USG image showing AFI at 34 weeks of gestation for the first patient. Amniotic fluid index (AFI) = 23.1cm

**Figure 7:**
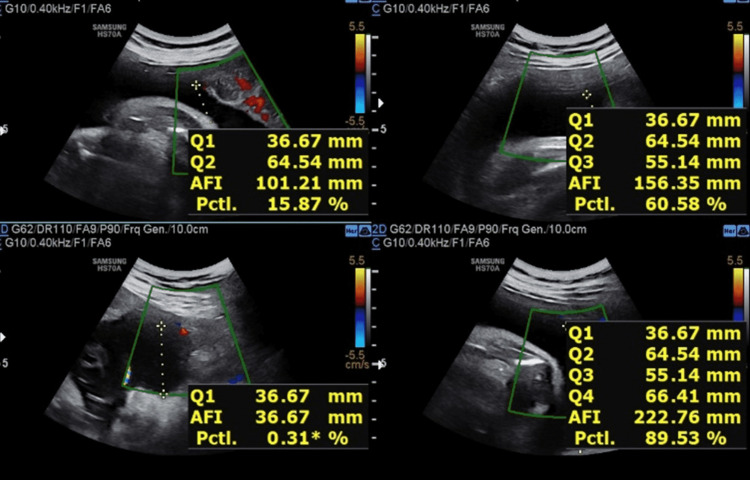
USG image showing AFI at 36 weeks of gestation for the first patient. Amniotic fluid index (AFI) = 22.2cm

**Figure 8:**
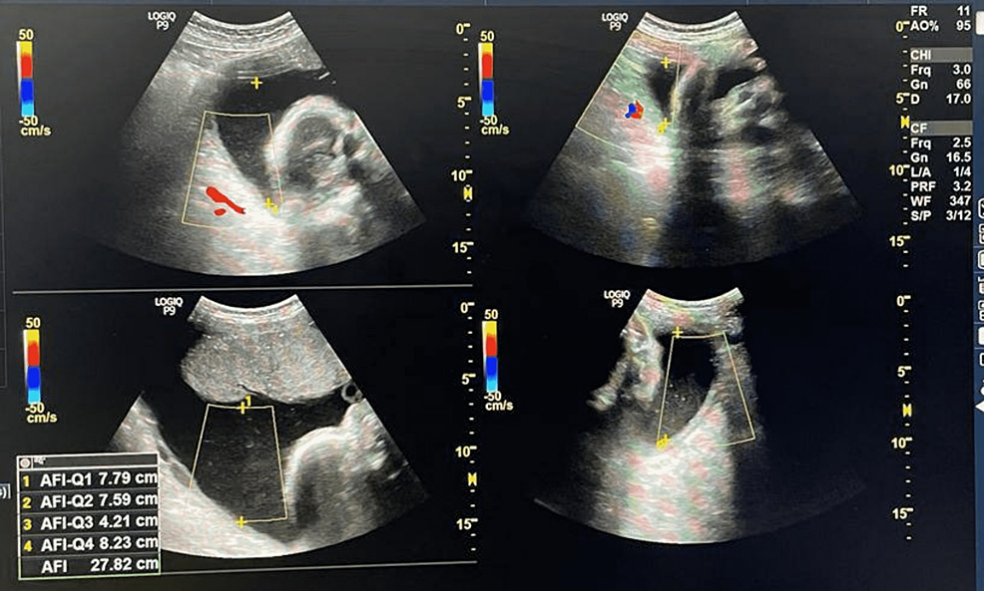
USG Image showing AFI at 28 weeks of gestation for the second patient Amniotic fluid index (AFI) = 27.8cm The patient was advised to restrict oral intake of sugar and blood samples were taken afterward.

**Figure 9:**
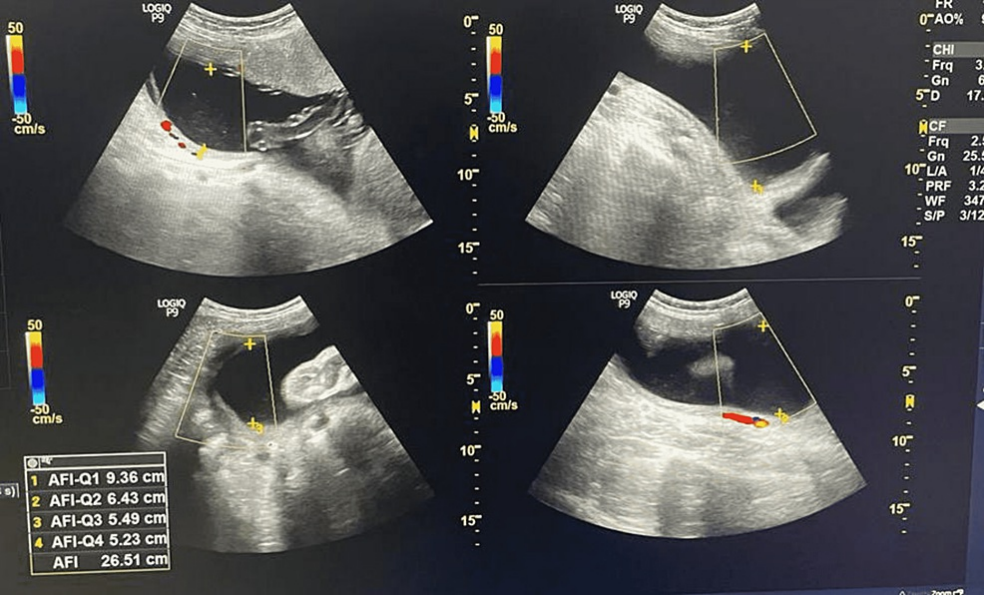
USG Image showing AFI at 32 weeks of gestation for the second patient Amniotic fluid index (AFI) = 26.5cm

**Figure 10:**
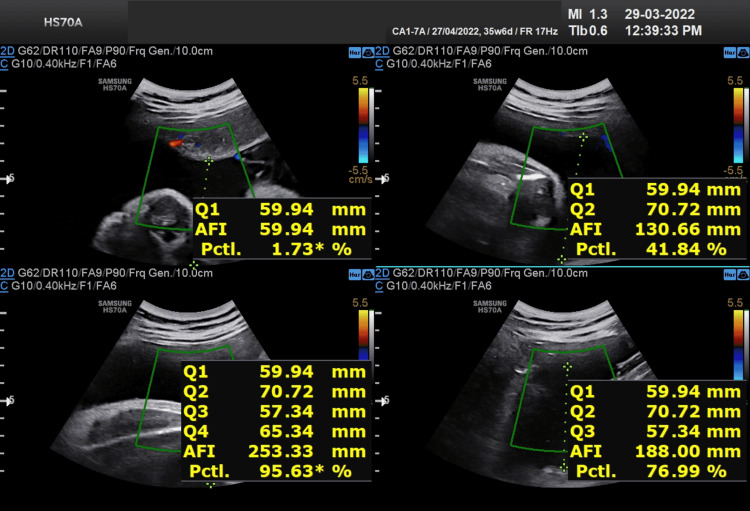
USG Image showing AFI at 34 weeks of gestation for the second patient. Amniotic fluid index (AFI) = 25.3cm

**Figure 11:**
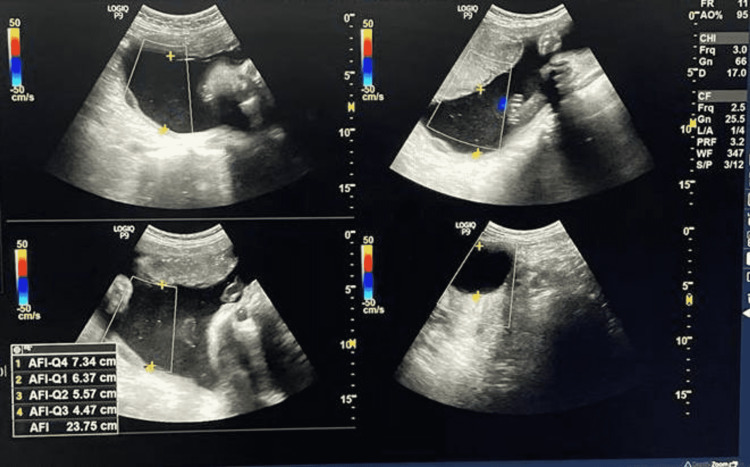
USG Image showing AFI at 36 weeks of gestation for the second patient Amniotic fluid index (AFI) = 23.7cm

Two radiologists (with more than five years of experience) reviewed the USG images independently. Both the radiologist who was involved in the study were purposefully blinded to clinical data to avoid any bias. A detailed evaluation of the images was done and changes were identified. The pregnant women with polyhydramnios (n=104) were asked to avoid oral intake of sugar until delivery, then a series of ultrasonography was performed at 28 weeks followed by 30 weeks, 32 weeks then at 36 weeks (Figures 4-11), and finally at term, we found that amniotic fluid index (AFI) could be brought under the normal range.

Table 3 shows the comparison of AFI in two different patients with respect to increasing gestational age after restricting oral intake of sugars beyond 28 weeks of gestation. 

**Table 3:** The comparison of AFI in two different patients with respect to increasing gestational age after restricting oral intake of sugars beyond 28 weeks of gestation.

AFI VALUES (cm) GESTATIONAL AGE	AFI VALUES (cm) FOR FIRST PATIENT	AFI VALUES (cm) FOR SECOND PATIENT
28 WEEKS OF GESTATIONAL AGE	25.7	27.8
32 WEEKS OF GESTATIONAL AGE	25.1	26.5
34 WEEKS OF GESTATIONAL AGE	23.1	25.3
36 WEEKS OF GESTATIONAL AGE	22.2	23.7

We concluded that there is a decrease in amniotic fluid index with increasing gestational age after restricting oral intake of sugar in pregnant females with polyhydramnios. We observed that the decline is more rapid towards the gestational age range of 28-36 weeks, whereas beyond 36 weeks the values stabilized at the end of pregnancy compared to the initial periods of gestation and showing that restriction of sugar for a prolonged duration of time can prevent complications related to gestational diabetes with polyhydramnios.

## Discussion

For all patients (n=104) who fulfilled the above-mentioned criteria, random blood sugar was taken and afterward, they were asked to avoid oral intake of sugar until delivery, then a series of ultrasonography was performed at 28 weeks followed by 30 weeks, 32 weeks then at 36 weeks, and finally at term. We measured amnionic fluid index and HbA1C in the serum of pregnant women along with glucose tolerance test, blood glucose fasting, and post-prandial and random blood glucose in pregnant women. The purpose of this study is to (i) evaluate AFI after avoiding oral intake of sugar in pregnant females with polyhydramnios, (b) evaluate the relationship between the amniotic fluid index and blood sugar levels in pregnancies, (c) predict gestational diabetes by amniotic fluid index much before serum blood sugar test, (d) determine whether polyhydramnios is associated with increased perinatal morbidity and mortality.

The danger of the subsequent obstetric complications is elevated while polyhydramnios is present and over - enlargement of the uterus [12-14]. Maternal dyspnea, early labor, early rupture of membranes, unusual fetal presentation, umbilical cord prolapse, postpartum hemorrhage, fetal macrosomia, hypertensive issue of being pregnant, and urinary tract infections. These dangers range from the severity and etiology of the polyhydramnios [15,16]. A prospective longitudinal study of normal singleton pregnancies lists the subsequent potential complications [17]: (a) increased rates of cesarean sections for fetal indications; (b) increased rates of admission to neonatal medical care units; (c) increased birth weight; and (d) decrease 5-minute Apgar scores. 

In a massive study of 85,000 pregnancies, of which 39,000 pregnancies had an elevated AFI, it had been observed that polyhydramnios became an unbiased danger aspect for perinatal mortality [18]. Small for fetal age (SGA) fetuses with polyhydramnios had the poorest prognosis.

The four-quadrant AFI for every polyhydramnios female was measured via a registered diagnostic sonographer using the technique initially started by Phelan et al. in which the maternal abdomen is split into four quadrants through the linea nigra as midline and the umbilicus to define the crossing X-axis. The biggest vertical pocket of fluid in each quadrant became measured and the sum of the four measurements became used as the AFI [19].

There has additionally been observational research performed in different international locations consisting of Germany, South Africa, the Netherlands, the United States, and Iceland. This research observed comparable institutions among sugar intake in being pregnant and extra maternal weight gain, both via examination of single or [20] vitamins meals groups [21], or nutritional styles that encompass an excessive consumption of introduced sugars [22-24].

Excessive consumption of introduced sugars at some stage in being pregnant is one of the nutritional variables that has been proven to be associated with the improvement of gestational diabetes mellitus (GDM). Research that used National Health and Nutrition Examination Survey statistics to study the nutritional styles of pregnant ladies and the danger of gestational diabetes observed that a weight loss plan characterized via way of means of excessive introduced sugar and organ meats; low fruits, greens, and seafood became the sample with the best danger for GDM [25].

Although little research has especially evaluated the outcomes of immoderate sugar intake on gestational weight gain, there's rising proof suggesting a nice association. To our knowledge, the biggest research to assess this relationship was a prospective cohort study of 46,262 women in Denmark. The authors observed that intake of introduced sugars at some stage in being pregnant became strongly and related to immoderate gestational weight gain. In contrast, a better protein-to-carbohydrate ratio became inversely related to weight gain [26]

Study limitations

It is tough to evaluate sugar consumption and its various types e.g., sucrose, fructose, and lactose, and its regional differences (for example, the United States regularly makes use of excessive fructose maize syrup in liquids, even as the equal beverage in different nations might also additionally incorporate sucrose).

There also are numerous types of opportunity sweeteners on the market, consisting of synthetic and herbal sweeteners, which can be used at special dosages and intensities, and might have differential outcomes in a secondhand sugar context. Quantifying opportunity sweetener consumption is likewise very tough due to the fact the precise quantities utilized in merchandise aren't normally reported.

There are limited human observational and controlled studies identifying associations of excessive sweetened food and beverage consumption with poor pregnancy outcomes. Animal research has demonstrated an increased incidence of gestational diabetes as well as altered maternal, fetal, and offspring metabolic function, although the long-term effects and the mechanism underlying these perturbations are ill-defined. This article aims to understand the role of early life sugar exposure in modifying the postnatal risk of disease in the offspring, focusing on fructose intake during pregnancy and in early postnatal life.

## Conclusions

A total of 104 patients that were enrolled in this study were pregnant females with polyhydramnios, a condition that is more common in the urban zone and in older pregnant females. In this study, we have concluded that the earliest and most sensitive predictor for gestational diabetes is the rise in the amniotic fluid index which could have been prevented by avoiding oral intake of sugar. Early prediction of gestational diabetes can be made by amniotic fluid index even before glucose concentration. We observed that by avoiding oral intake of sugar, the amniotic fluid index drops down in pregnant females with polyhydramnios. We also observed that the decline is more rapid towards in the gestational age range of 28-36 weeks, whereas beyond 36 weeks the values stabilized at the end of pregnancy compared to the initial periods of gestation and showing that restriction of sugar for a prolonged duration of time can prevent complications related to gestational diabetes with polyhydramnios.
